# Prediction of risk factors for preoperative deep vein thrombosis in patients with pelvic fracture

**DOI:** 10.3389/fsurg.2025.1585460

**Published:** 2025-04-28

**Authors:** Yufen Chen, Jingyuan He, Xia Pan

**Affiliations:** Department of Orthopaedics, The First Affiliated Hospital of Soochow University

**Keywords:** pelvic, prediction model, risk factor, deep vein thrombosis (DVT), preoperative

## Abstract

**Objective:**

This study aims to develop a preoperative risk assessment tool for deep vein thrombosis (DVT) in pelvic fracture patients, offering evidence-based guidance for surgeons.

**Methods:**

A cohort of 400 pelvic fracture patients was analyzed. Ten candidate predictors were initially identified via LASSO regression from 25 clinical variables. Four independent risk factors—emergency abdominal surgery, Injury Severity Score (ISS), serum creatinine levels, and aspartate aminotransferase (AST)—were subsequently incorporated into a multivariate logistic regression model. A nomogram was developed using R software, with calibration accuracy assessed via the rms package and clinical utility evaluated through decision curve analysis (DCA) using the ggDCA package.

**Results:**

The final model demonstrated excellent discriminative ability, with area under the curve (AUC) values of 0.88 (95% CI: 0.81–0.93) in the training cohort and 0.88 (95% CI: 0.80–0.95) in the validation cohort. Calibration curves confirmed strong alignment between predicted and observed DVT probabilities, while DCA highlighted the nomogram's clinical applicability across a wide risk threshold range.

**Conclusion:**

The validated nomogram provides a reliable preoperative tool for stratifying DVT risk in pelvic fracture patients. By enabling early identification of high-risk individuals, this model supports targeted prophylactic interventions, ultimately enhancing perioperative safety and patient outcomes.

## 1 Introduction

Pelvic fractures are often caused by high-energy violence, such as traffic accidents and falls from high places. Clinical manifestations include pain, swelling, limited mobility, and deformity. Pelvic fractures can lead to serious complications, such as excessive bleeding, internal damage, and nerve damage (1). Adult pelvic fractures accounted for 3.87% of adult fractures in the same period (2). The incidence of early postoperative deep vein thrombosis (DVT) in patients with pelvic fractures can be as high as 12%–40%. In the absence of thromboprophylaxis, the incidence of Venous thromboembolism (VTE) in patients with pelvic fractures can be as high as 30% (3).

VTE (4) is an abnormal clotting of blood in a vein that blocks the venous lumen and causes dysregulation of venous blood, mainly including deep vein thrombosis (DVT) and pulmonary embolism (PE). DVT is a common complication after trauma (5), and it is easy to occur in patients with pelvic and lower limb fractures. A thrombus attached to the wall of a deep vein can cause adverse events such as chest pain, hypoxia, and even sudden death if it breaks off and blocks the main branches of the pulmonary artery.

Patients with unstable pelvic fractures are often complicated with potentially life-threatening conditions such as multiple injuries, sepsis, and hemorrhagic shock, and may be at high risk for DVT in the orthopedic ward (6). In the clinical practice of predicting thrombosis following pelvic fractures, numerous challenges impede accurate assessment. The complex anatomical structure of the pelvis, with its intricate network of blood vessels, makes it arduous to precisely detect early signs of thrombosis. Moreover, the diverse injury mechanisms and concomitant injuries in patients with pelvic fractures introduce significant variability in clinical manifestations, further complicating the diagnostic process. Despite these difficulties, the prediction of thrombosis after pelvic fractures holds crucial clinical relevance. The objective of this study was to investigate the incidence of DVT in patients with pelvic and lower limb fractures before surgery and to explore the independent risk factors for DVT. Moreover, it can provide more information for the early identification of high-risk groups of DVT and the prevention of DVT.

## 2 Material and methods

### 2.1 Patients selection

This study was retrospective and conducted from January 2016 to December 2023. Clinical data of patients admitted to the orthopedic department of a local hospital were analyzed. Patients with pelvic fractures caused by trauma, with or without lower extremity fractures were included in the study. The time duration from injury to operation was less than 2 weeks. The ultrasound examination of the lower limb vessels was completed before internal fixation. Patients with pelvic fractures caused by trauma, patients with age no less than 18 years old, and patients with time from injury to operation less than 2 weeks were included in the study. Patients with pathological fracture, patients receiving anti-coagulation or anti-platelet therapy before fracture, patients complicated with tumor or blood system disease, patients presenting DVT before fracture, and patients with incomplete clinical data were excluded from the study.

### 2.2 Data collection

The clinical data of patients were extracted from the electronic medical record system of the hospital information database. Demographic data, including sex, age, and body mass index (BMI) were recorded. Diagnostic information, such as the cause of injury, site of the fracture, injury severity score, diabetes, hypertension, and blood transfusion was recorded. Laboratory data within 24 h of hospitalization, coagulation parameters, liver and kidney function were also recorded. According to the results of the lower extremity arteriovenous color ultrasound before plevic surgery, the subjects were divided into the DVT group and the non-DVT group.

### 2.3 Statistical analysis

SPSS 19.0 software and R language were used for data processing and statistical analysis. Measurements with normal distribution were analyzed by the mean *t*-test and the independent samples *t*-test for comparison between the two groups. The Mann–Whitney *U*-test was used for the comparison between the two groups of non-normally distributed measures. Count data were expressed as frequency (percentage) *n* (%), and the χ^2^ test was used for comparison between the two groups. The potential risk factors between the DVT and non-DVT groups were screened by Lasso regression and then analyzed by multivariate logistic regression. Based on the independent risk factors selected by multifactorial logistic regression, a nomogram prediction model was constructed. The discriminability of the prediction model was evaluated by plotting the receiver operating characteristic (ROC) curves of the training and validation sets. Bootstrap (BH = 1,000) was used for internal verification. The calibration of the predictive model was evaluated by plotting calibration curves. The clinical applicability of the predictive models was evaluated by decision curve analysis (DCA). 70% of the data were randomly extracted and used as the validation set, and the C-index was calculated. *P* < 0.05 was considered as statistically significant difference.

## 3 Results

### 3.1 Sampling and data collection

**Figure 1:**
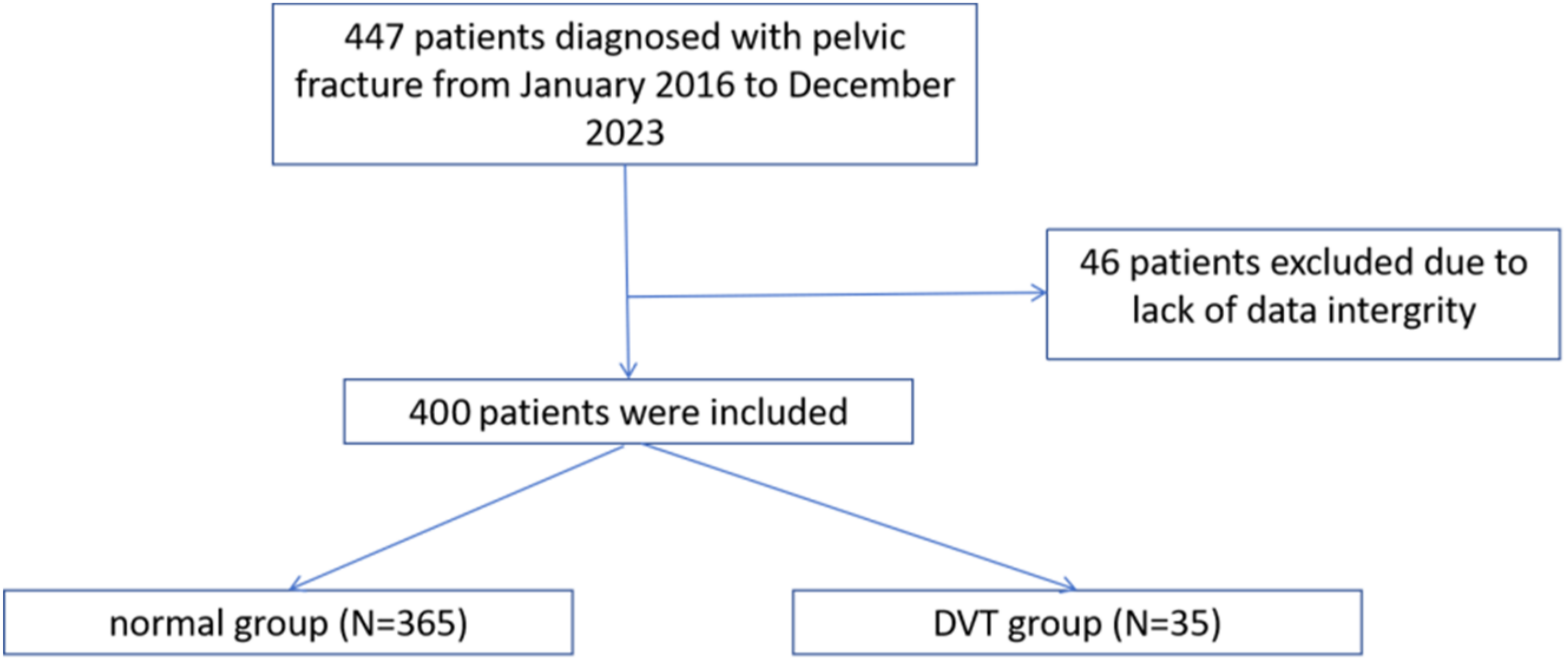
The flow chart of the present study.

The selection process of research objects was shown in Figure 1. The clinical data of 447 patients with pelvic fractures were analyzed. Forty-six patients were excluded due to a lack of data integrity. A total of 400 patients were included in the study, 35 of whom developed DVT.

### 3.2 Comparison of different variables between patients with and without DVT

The comparison of clinical data between the DVT group and the non-DVT group is shown in Table 1. There were statistically significant differences between the two groups (*P* < 0.05), in terms of the location of the fracture, combination with emergency abdominal surgery, ISS score, serum creatinine, white blood cell count, and AST.

**Table 1:** The comparison of clinical data between normal and DVT groups.

Clinical variables	Normal	DVT	p.overall
	*N* *=* *365*	*N* *=* *35*	
Age (year)	53.4 (14.1)	55.3 (15.1)	0.47
Gender			
Male	212 (58.1%)	17 (48.6%)	0.36
Female	153 (41.9%)	18 (51.4%)	
BMI (kg/m^2^)	23.9 (4.56)	23.0 (4.38)	0.25
Admission time (h)	230 (102)	257 (103)	0.14
Fracture site			
1	140 (38.4%)	16 (45.7%)	**0** **.** **01**
2 femourcture	186 (51.0%)	9 (25.7%)	
3	39 (10.7%)	10 (28.6%)	
ISS score	24.5 (5.75)	26.9 (5.95)	**0** **.** **02**
Combination with other surgery			
No	355 (97.3%)	29 (82.9%)	**0** **.** **01**
Yes	10 (2.74%)	6 (17.1%)	
Blood loss (ml)	282.2 (319)	359.3 (326)	0.19
Diabetes			
No	247 (67.7%)	21 (60.0%)	0.46
Yes	118 (32.3%)	14 (40.0%)	
Hypertension			
No	258 (70.7%)	22 (62.9%)	0.33
Yes	107 (29.3%)	13 (37.1%)	
Smoking			
No	270 (74.0%)	21 (60.0%)	0.08
Yes	95 (36.0%)	14 (40.0%)	

In terms of fracture sites, patients were divided into three categories: pelvic only (1), pelvic with femur fracture (2), and pelvic with lower extremity fracture other than femur (3). Bold color indicate statistical significance.

### 3.3 LASSO regression to filter the candidate variables

To filter out possible risk factors of DVT, a LASSO regression model was created to reduce 25 variables to 10 potential risk factors, including BMI, serum cholesterol, combination with emergency abdominal surgery, ISS score, serum creatinine, neutrophil counts, INR, FIB, lymphocyte count and AST. Finally, four risk factors filtered both by LASSO regression and statistical comparison were used as potential risk factors in the next step, which are a combination of emergency abdominal surgery, ISS score, serum creatinine, and AST.

### 3.4 The establishment of multi-logistic regression model

**Figure 2:**
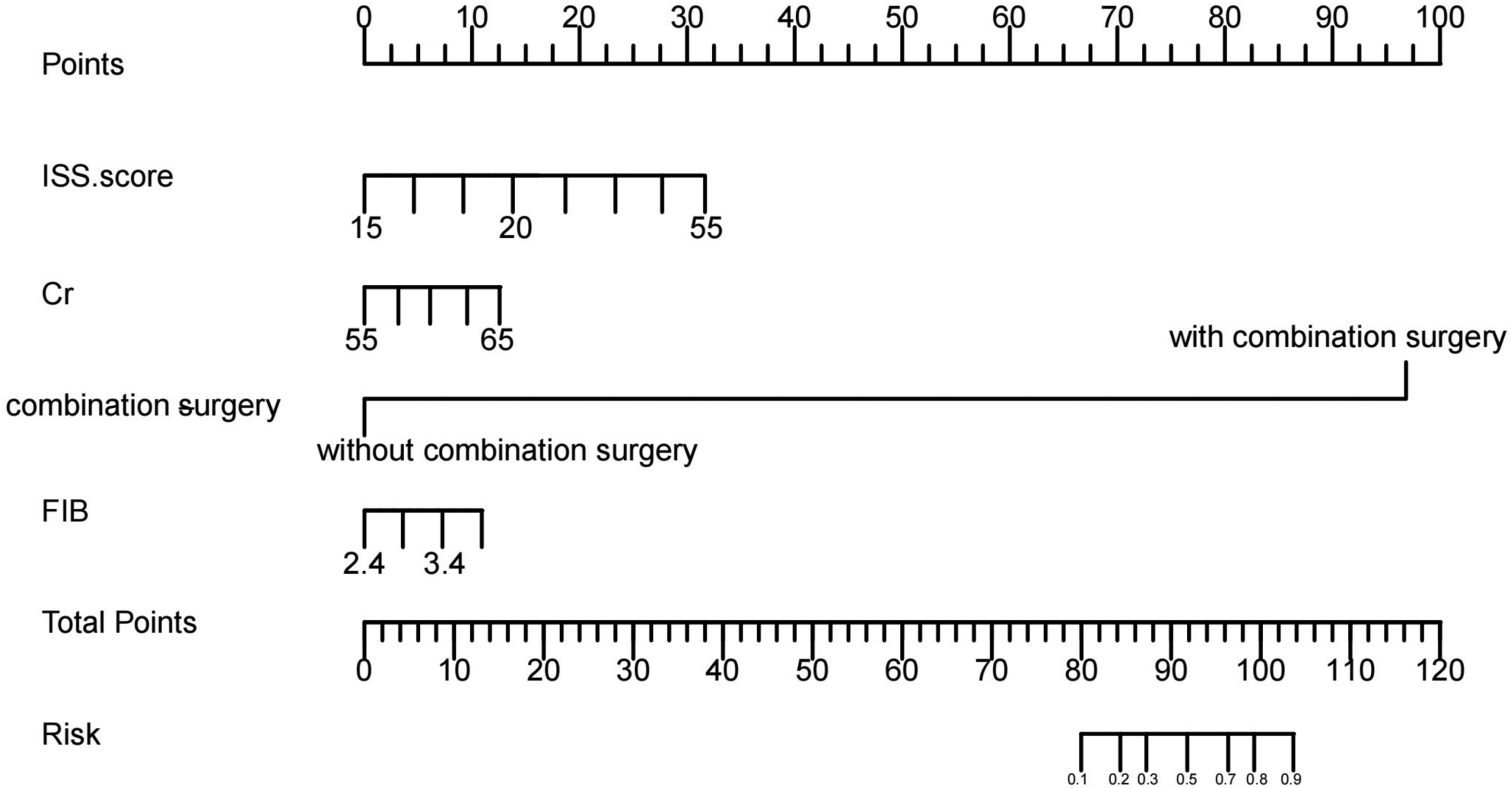
Nomogram of risk predictors based on logistic regression. The DVT nomogram was developed based on the training data set, with creatinine, complicated with other surgery, ISS score, and FIB plotted in the figure.

The five potential risk factors were included in a multivariable logistic regression model, as shown in Table 2. Further, a nomogram was plotted to visualize the contribution of each risk factor to the occurrence of DVT, as illustrated in Figure 2.

**Table 2:** The comparison of laboratory data between normal and DVT group.

Laboratory variables	Normal	DVT	p.overall
	*N* *=* *365*	*N* *=* *35*	
Cholesterol (g/L)	4.64 (2.35)	5.39 (2.64)	0.11
Triglyceride (g/L)	1.64 (1.43)	2.03 (1.31)	0.11
Creatinine (μmol/L)	60.2 (0.58)	59.5 (0.57)	**<0** **.** **01**
Albumin (g/L)	40.1 (19.7)	38.5 (3.71)	0.21
Hemoglobin (g/L)	132 (60.8)	127 (18.4)	0.26
White blood cell (×10^9^/L)	12.3 (52.5)	6.49 (1.96)	**0** **.** **04**
Platelet (×10^9^/L)	225 (20.3)	225 (19.3)	0.86
FIB (g/L)	3.06 (0.33)	3.16 (0.37)	0.14
APTT (s)	10.9 (0.80)	11.1 (0.81)	0.33
INR	1.07 (0.07)	1.05 (0.07)	0.12
D-dimer (μg/L)	0.54 (0.15)	0.54 (0.15)	0.80
Neutrophil (×10^9^/L)	4.95 (0.75)	4.75 (0.79)	0.17
Lymphocyte (×10^9^/L)	1.99 (0.46)	2.16 (0.34)	0.17
ALT (U/L)	46.7 (14.5)	45.4 (11.8)	0.53
AST (U/L)	54.7 (22.2)	61.2 (22.1)	**0** **.** **01**

ALT, alanine aminotransferase; AST, aspartate aminotransferase. Bold color indicate statistical significance.

### 3.5 Assessment of the nomogram established

**Figure 3:**
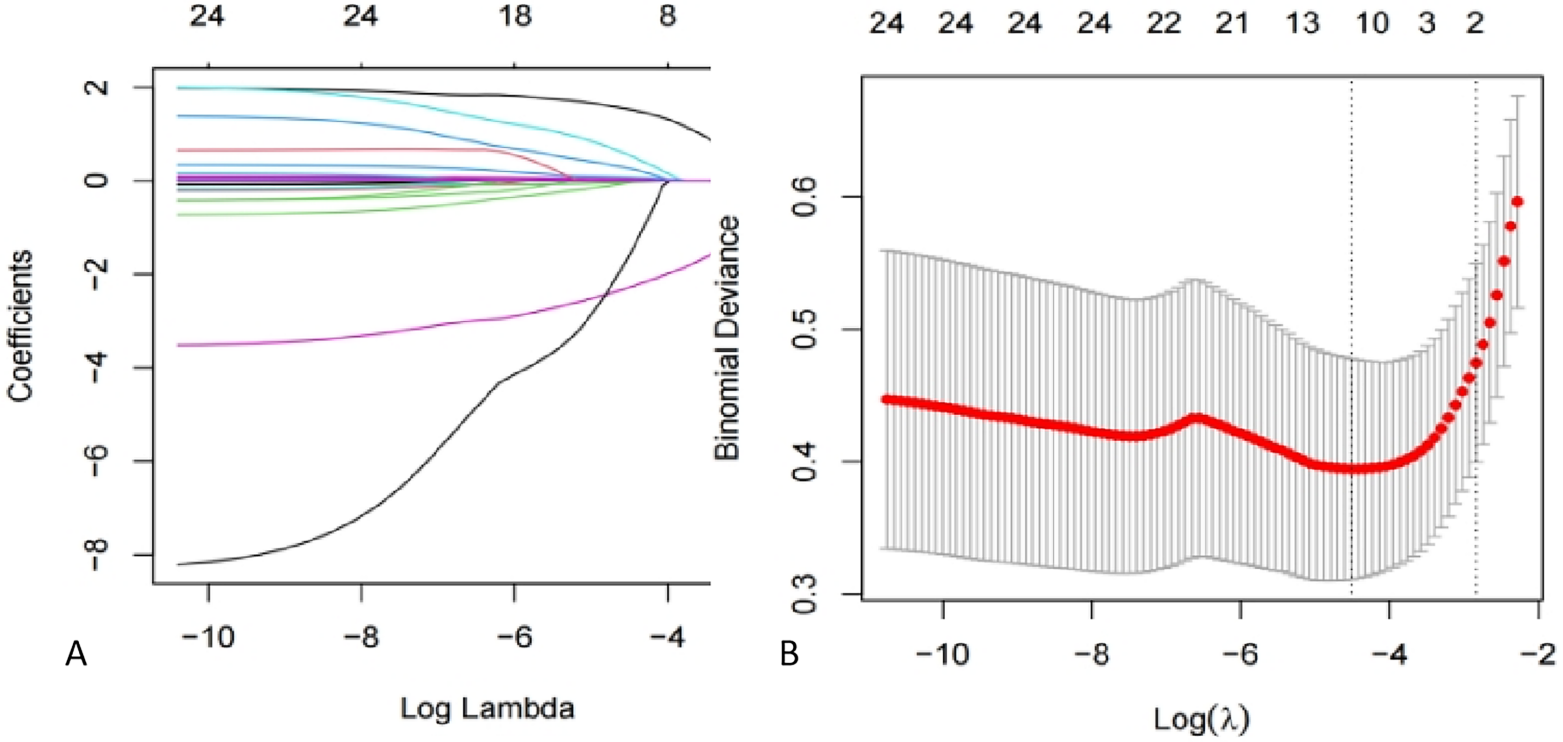
LASSO regression modeling to screen for potential risks. **(A)** Ten-fold cross-validation was used to select the optimal parameter of the LASSO model with the lowest criteria. **(B)** Plot of partial likelihood deviation. Vertical dashed lines are drawn at the optimal values using the minimum criterion and the 1 SE of the minimum criterion (1-SE criterion). LASSO, least absolute shrinkage, and selection operator; SE, standard error.

**Figure 4:**
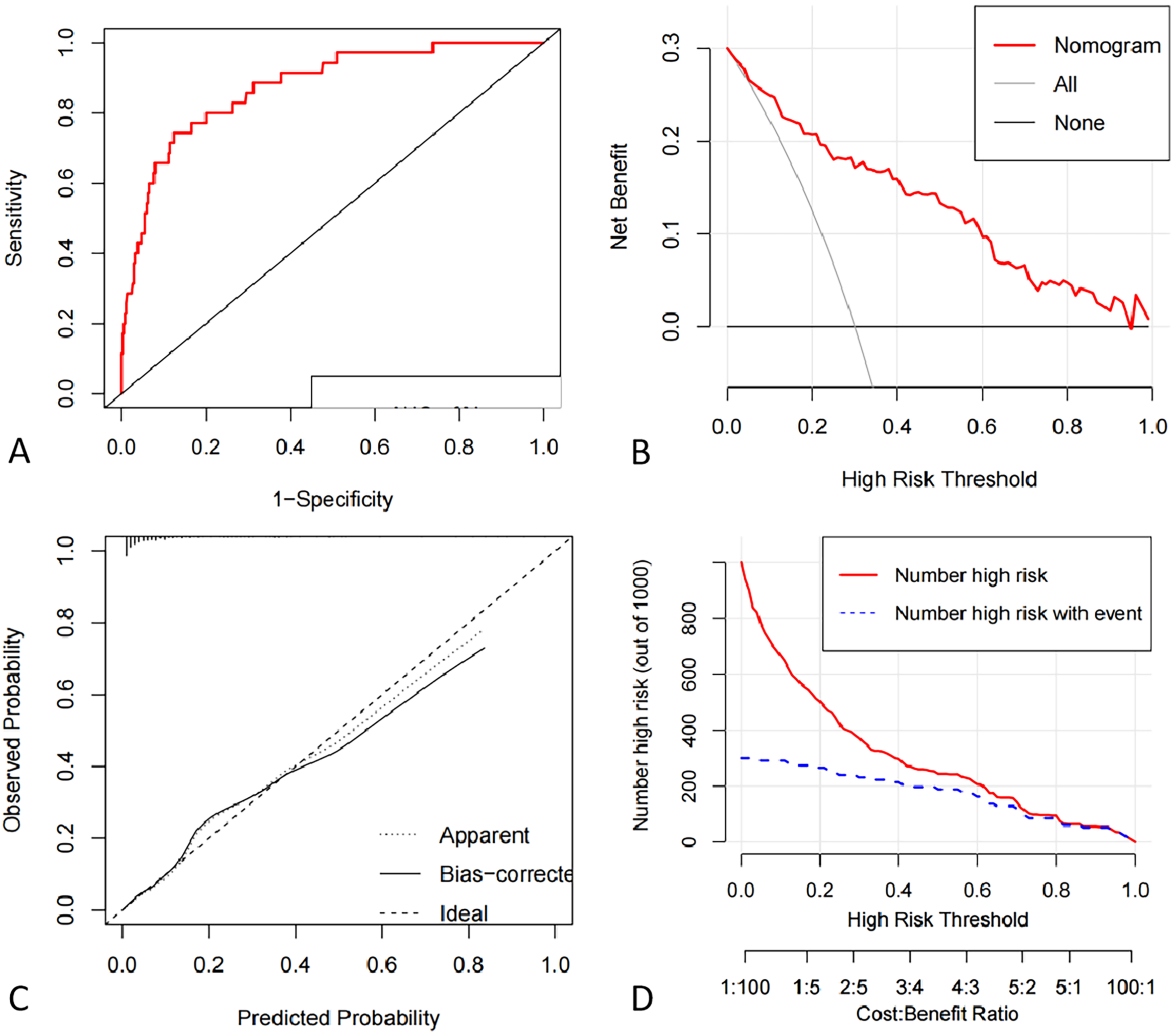
**(A)** ROC curve of deep vein thrombosis risk profile model for pelvic and lower limb fractures. **(B)** Decision curve analysis for the intraoperative hypothermia nomogram. The *Y*-axis indicate net benefits. The thin solid line represents the hypothesis that hypothermia occurred in all patients. The thick solid line indicates the hypothesis that no patient developed hypothermia. The DCA curve analysis showed that the net benefit of the intervention was positive when the probability of DVT was within a certain range between 0.36–0.96. **(C)** Calibration curves of the nomogram prediction using training data set. The Apparent line means the performance of the self-training. The bais-corrected line means the performance of the model trained by repeated self-sampling of the samples, which corrects the overfitting situation. **(D)** Clinical Impact Curve. When the threshold value is greater than 0.6, the model prediction and the actual occurrence are highly matched, and the clinical prediction efficiency is high.

As present in Figures 3A,B, the ROC curve of the nomogram suggested good discrimination ability [AUC = 0.87 95% CI (0.81, 0.93)] and the calibration curve of the nomogram with Hosmer Lemeshow goodness of fit test showed well calibrated (*p* > 0.05). DCA curve analysis showed that the training set received a positive net benefit from the intervention when the probability of DVT was 4%–95% calculated by the nomogram model. Clinical Impact Curve (CIC) indicated that when the threshold value was greater than 0.6, the model prediction and the actual occurrence were highly matched, and the clinical prediction efficiency was good, as shown in Figure 4.

### 3.6 Internal validation

**Figure 5:**
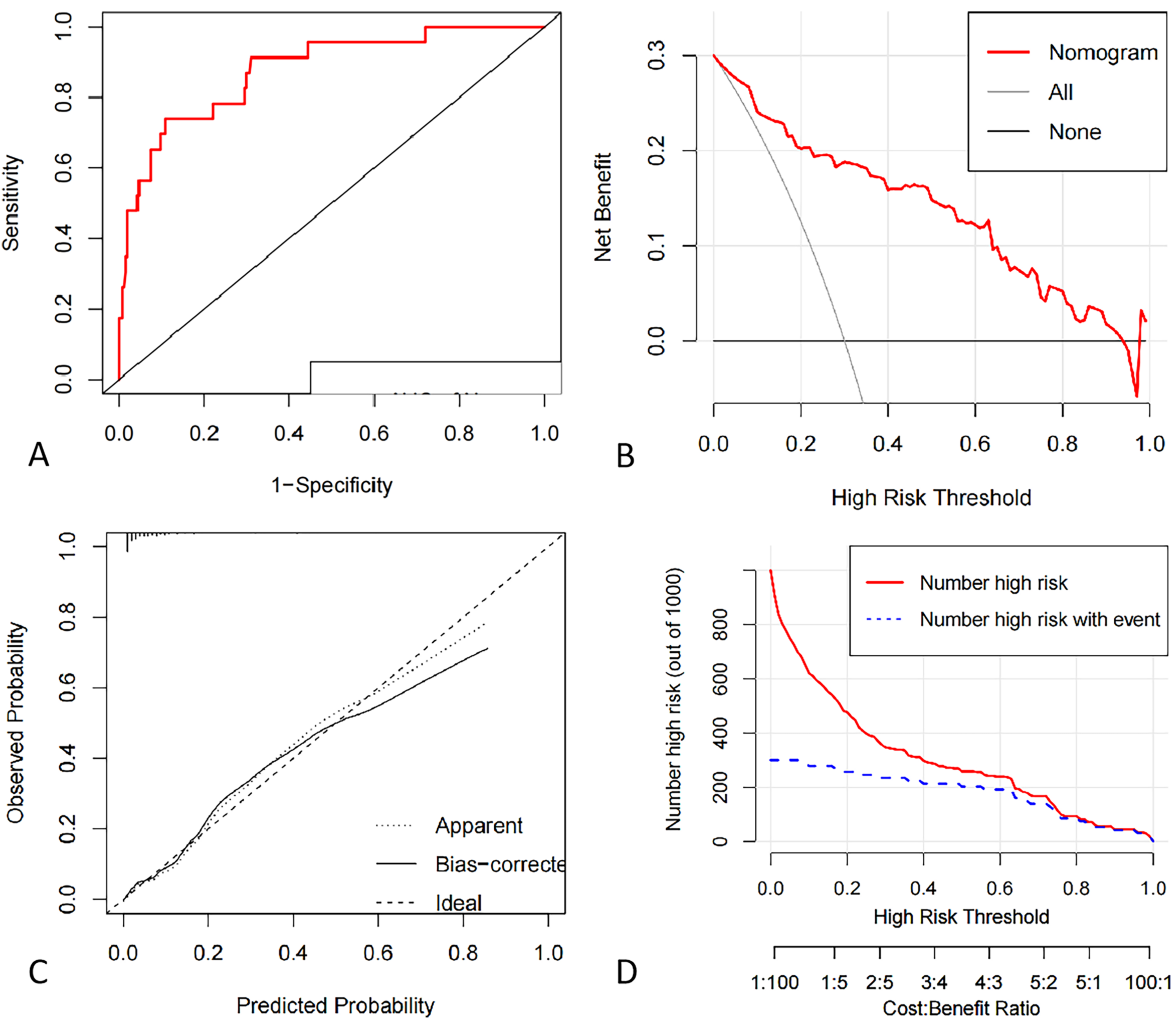
**(A)** ROC curve of deep vein thrombosis risk profile model for pelvic and lower limb fractures based on validation dataset. **(B)** Decision curve analysis for the intraoperative hypothermia nomogram. **(C)** Calibration curves of the nomogram prediction using validation data set. **(D)** Clinical Impact Curve.

The internal validation C-index was 0.88 (95% CI: 0.80–0.95), indicating that the nomogram had good discrimination and good predictive ability for predicting the probability of DVT occurrence, as shown in Figure 5.

## 4 Discussion

Deep vein thrombosis (DVT) frequently co-occurs with pelvic fractures, with such injuries substantially elevating thrombotic risk (7). Conversely, DVT exerts a detrimental influence on clinical decision-making by complicating treatment strategy selection, increasing procedural complexity, and prolonging rehabilitation timelines for pelvic fracture patients (8). Furthermore, DVT significantly amplifies the likelihood of life-threatening complications, including pulmonary embolism. Prophylaxis against DVT prior to pelvic fracture surgery constitutes a critical objective in perioperative care (9).

In this retrospective cohort study, we analyzed clinical data from 401 patients with pelvic or lower extremity fractures. Through LASSO regression and comparative statistical analysis, four independent predictors of DVT were identified: emergency abdominal surgery, Injury Severity Score (ISS), serum creatinine levels, and aspartate aminotransferase (AST) concentration. The derived nomogram demonstrated robust discrimination in predicting DVT occurrence, achieving area under the curve (AUC) values of 0.88 (95% CI: 0.81, 0.93) in the training cohort and 0.876 (95% CI: 0.80–0.95) in the validation cohort. Calibration analysis revealed strong model reliability, with calibration curves showing excellent alignment between predicted probabilities and observed outcomes. Decision curve analysis (DCA) further confirmed the model's clinical utility, demonstrating positive net benefit across a clinically relevant probability threshold range (0.36–0.96).

It is well established that trauma severity increases the risk of VTE, and it is challenging to accurately assess trauma severity. In terms of the mechanism of bone injury, it is confirmed that high-energy bone injury (such as falls above 1.22 m, traffic accidents, direct impact, etc.) is an independent risk factor for preoperative VTE (10). The degree of trauma is also reflected in shock, soft tissue injury, elevated serum lactic acid, liver, spleen or spinal cord injury, need for blood transfusion, Glasgow score, ISS score, etc. (11). The degree of bone trauma is reflected in the mechanism of bone injury and the patient's internal environment, and the accurate assessment of the severity needs further research. In this study, the mean ISS score in normal group was significantly lower than that in DVT group (*P* = 0.02). ISS is commonly used in clinical work to assess the severity of trauma patients. High ISS score indicates severe trauma, which can lead to severe internal environment and coagulation dysfunction, release of a large number of inflammatory factors into the blood and extensive vascular endothelial injury, thus promoting the formation of DVT (12).

Deep vein thrombosis (DVT) is a significant complication of abdominal surgery, with an incidence of 15%–30% in non-prophylactic major general surgery patients (13). This study highlights emergency abdominal surgery as an independent DVT risk factor in pelvic fracture patients, mediated through synergistic mechanisms (14–16): Surgical trauma disrupts vascular endothelial integrity, exposing thrombogenic subintimal collagen and activating coagulation, while the systemic stress response amplifies clotting factors and suppresses fibrinolysis. Intraoperative blood loss and fluid shifts further concentrate coagulation components, creating a hypercoagulable state. Anesthesia-induced muscle relaxation eliminates lower limb venous pumping, causing vascular dilation and blood stasis, compounded postoperatively by immobilization from abdominal drains, bandages, and bed confinement. Prolonged procedures with intestinal paralysis may mechanically compress abdominal veins due to visceral dilation, exacerbating venous congestion. These perioperative factors—vascular injury, hypercoagulability, venous stasis, and mechanical obstruction—collectively drive thrombus formation in this high-risk population.

Fibrinogen is a key clotting factor that significantly contributes to deep vein thrombosis (DVT) risk (17). As a central protein in the clotting cascade, it is converted by thrombin into fibrin to form clots and also impacts platelet aggregation and blood viscosity regulation. Elevated fibrinogen levels promote thrombosis by increasing blood viscosity and enhancing platelet aggregation. Studies have shown that for every 1 g/L increase in fibrinogen levels, the risk of DVT increases by approximately 2–3 times (18). Fibrinogen is an acute phase reactant whose levels are significantly elevated during inflammation. This inflammatory state further increases the risk of DVT by promoting endothelial damage and clotting activation (19). Patients with certain hereditary hyperfibrinogenemia (e.g., mutations in the fibrinogen gene) are more prone to thrombotic events, and polymorphisms in the fibrinogen gene have been associated with DVT risk (20). Elevated fibrinogen levels work synergistically with other risk factors, such as obesity, smoking, diabetes, and high blood pressure, to further increase the risk of DVT.

Elevated creatinine is a hallmark of renal insufficiency, and patients with renal insufficiency often have abnormal coagulation, inflammation, and endothelial dysfunction, which can increase the risk of thrombosis. Studies have shown that hypercoagulability in patients with renal insufficiency is closely related to the occurrence of DVT (21). Elevated serum creatinine is an important indicator of CKD. Patients with CKD often have hypercoagulability, inflammation, and oxidative stress, which promote thrombosis. Patients with elevated creatinine often have high blood pressure, diabetes, and cardiovascular disease, which themselves increase the risk of DVT (22).

This study has several limitations. Firstly, it is a single-center retrospective study, which may inevitably introduce bias due to incomplete consideration of factors affecting DVT. Secondly, vascular ultrasound, used for DVT diagnosis, is not the “gold standard” and may lead to diagnostic bias. Additionally, the small number of patients in some groups, including only 16 patients undergoing emergency abdominal surgery, may affect the accuracy of the results despite multi-factor analysis correction. The lack of external validation for the developed clinical predictive model also limits the assessment of its applicability and stability across different clinical settings. These findings need confirmation through multi-center, large-sample, prospective studies, and future work will focus on external validation.

## 5 Conclusion

The nomogram developed for preoperative prediction of deep vein thrombosis (DVT) demonstrated strong predictive accuracy. This evidence-based tool enables clinicians to systematically evaluate patients’ thrombotic risks prior to surgical interventions.

## Data Availability

The raw data supporting the conclusions of this article will be made available by the authors, without undue reservation.
